# Co‐Design or Faux‐Design? Reflections on Co‐Designing Safe Spaces for People in Emotional Distress or Suicidal Crisis in Australia

**DOI:** 10.1111/hex.70379

**Published:** 2025-08-11

**Authors:** Erin Oldman, Michelle Banfield, Heather Lamb, Erin Stewart, Helen Tosin Oni, Benn Miller, Mel Giugni, Alyssa R. Morse, Scott J. Fitzpatrick

**Affiliations:** ^1^ Roses in the Ocean Brisbane Australia; ^2^ Centre for Mental Health Research, National Centre for Epidemiology and Population Health The Australian National University Canberra Australia; ^3^ The ALIVE National Centre for Mental Health Research Translation The Australian National University Canberra Australia; ^4^ Independent Researcher and Lived Experience Advocate Canberra Australia; ^5^ College of Medicine and Public Health Flinders University Adelaide Australia; ^6^ Sonder Adelaide Australia; ^7^ Towards Zero Suicides Initiatives South Western Sydney Local Health District Sydney Australia

**Keywords:** co‐design, lived experience, mental health, safe spaces, service users, suicide

## Abstract

**Background:**

The past decade has seen an increase in non‐clinical ‘safe spaces’ for those experiencing suicidal crisis or distress. Integrating service user perspectives through co‐design is increasingly recognised as essential for the design of these services to meet user needs. Operationalising genuine co‐design practices involving diverse stakeholders in local contexts remains underdeveloped, and research remains limited.

**Objective:**

Drawing on co‐design participants' experiences, this study evaluates how co‐design processes influenced the design and implementation of safe space models in Australia.

**Design:**

A mixed‐methods design was used to analyse survey, interview and documentary data for six safe space co‐design projects. Thematic synthesis and triangulation were applied to develop overarching themes.

**Setting and Participants:**

Key partners, steering committee members and lived experience representatives involved in the co‐design and implementation of the six sites participated in surveys and interviews.

**Results:**

Power imbalances between health services staff and lived experience representatives were key barriers to genuine engagement, alongside tokenistic co‐design, or ‘faux‐design’. Despite these challenges, all participants reflected positively on their involvement.

**Conclusions:**

Effective co‐design requires trust, transparency, power sharing, sufficient resourcing and sustained lived experience engagement throughout the project life cycle. Health service providers must assess their capacity for authentic engagement before attempting co‐design. Future co‐design initiatives should focus on ensuring that lived experience input is not lost during implementation. Future research should explore how to support and sustain this engagement throughout all project phases.

**Patient or Public Contribution:**

People with lived experience of emotional distress and/or suicidal crisis, including academic researchers, health, community service and peer workers, carers, and advocates were involved in this study. All authors identify as people with lived experience, from both academic and non‐research backgrounds.

## 1 Introduction

Safe spaces, also called safe havens, offer safe, accessible, non‐clinical support for people experiencing suicidal thoughts or emotional distress. First implemented in the United Kingdom as an alternative to hospitals and emergency departments (EDs), Australia's first safe space was established in 2018 [1]. Since then, government investment has rapidly expanded its development, with several models currently being trialled.

The emergence of safe spaces has coincided with growing recognition among policymakers, practitioners, researchers and service user networks of the value of integrating lived experience perspectives into health service design. Co‐design approaches that meaningfully engage health service users are internationally recognised as best practice in developing high‐quality mental health and suicide prevention services, with significant contributions from health systems in the United Kingdom, Canada and the United States [2, 3, 4, 5]. Effective co‐design is considered more ethical, democratic and capable of producing positive, lasting changes in service users' experiences and health outcomes than traditional methods [6, 7]. Since 2020, Australian policy has increasingly mandated the inclusion of service users' needs and ideas for change [1, 8, 9].

While research on co‐design in healthcare has a relatively established history, few studies have focused specifically on the co‐design of safe space models, the successes and challenges of these processes, or how they influence the implementation of mental health services [10, 11]. This paper builds on the emerging body of work in mental health by examining the experiences and expectations of those who co‐designed six safe space service models in Australia. We contribute new insights into how co‐design practices affected participants and shaped service development and delivery.

## 2 Materials and Methods

### 2.1 Setting and Participants

The study was conducted across six safe spaces in three jurisdictions: the Australian Capital Territory (ACT), New South Wales (NSW) and South Australia (SA). Participants were aged 16 years and over and had been active as a key partner, steering committee member or co‐design participant and contributed to the co‐design, set‐up, implementation or management of one or more of the safe spaces studied.

### 2.2 Ethics

The study ethics and consent procedures were approved by the ACT Health Human Research Ethics Committee (Reference Number 2022.ETH.00043). Informed consent was obtained from all participants.

### 2.3 Research Design

The investigation of co‐design partners' perspectives on co‐design practices and processes was a sub‐study of the ‘Co‐Creating Safe Spaces’ project [12]. Broadly, the overarching research project aimed to assess the feasibility of safe space models in community settings as alternatives for people who might usually present to the ED or avoid services due to past negative experiences. The project involved a range of lived experience, research and health services partners, and co‐creation was built into all aspects of the research. This co‐design study was led by lived experience researchers who designed the study, collected and analysed the data, and authored the paper. Author M.B. was a member of the steering committee for one site, but the researchers were not involved in co‐design at any of the six sites. Our viewpoint as researchers is shaped by our diverse lived experiences, which inform the lens through which we interpret the data and discuss the findings.

We employed a convergent mixed‐methods design, collecting and analysing quantitative and qualitative data in parallel [13]. The types of data generated include survey, interview and organisational documentary data. Bespoke online surveys and semi‐structured interviews were co‐designed with our study co‐creation partners, who ran service co‐design processes. Data captured participants' experiences of co‐designing the new service models, the impacts of the co‐design process, and their perceptions of the implementation of the co‐design plans. Documentary data provided background and context, offering insights into different co‐design processes and safe space models, and aiding in evaluating fidelity during implementation. It also helped verify findings and contextualise survey and interview data [14]. The data collection methodology ensured alignment between qualitative and quantitative questions, enabling effective comparison and integration of data outputs [13]. Sample size decisions were made throughout the project based on interpretative, situated and pragmatic judgements about time, resources and the aims of the research [15].

### 2.4 Data Collection

We requested access to co‐design‐related documents from project partners, and with the consent of those involved. All health service delivery personnel and community members involved in the co‐design were invited through a recruitment email to participate in the online survey, an interview or both. Data were collected between February 2023 and January 2024.

The 39‐item online survey collected quantitative data through 32 Likert‐type and multiple‐choice questions (see Supporting Material) and qualitative data through seven open‐response questions. Data comprised participant roles, lived experience, co‐design experiences and implementation outcomes. No identifiable data were collected from survey participants; therefore, it was not known if participants completed both a survey and an interview. Interview questions elicited qualitative data through semi‐structured open‐response questioning and focused on participation, expectations, process and impacts (see Supporting Material). Interview recordings were auto‐transcribed using Microsoft Word and Zoom. Final transcription was then performed (verbatim) by the co‐investigator team and de‐identified for subsequent analysis. In keeping with the principles of lived experience research established by the team, interview participants were provided the choice to nominate their attribution in the research outputs in one of three ways: full name, pseudonym or confidential/no attribution, and were given the opportunity to review their transcript. Quotes are attributed by name where requested; open‐ended survey responses are attributed by role. Documentary data included 12 planning and reporting documents from 4 safe space sites.

### 2.5 Data Analysis

We conducted quantitative assessments of Likert‐type and multiple‐choice survey responses, as well as qualitative assessments of open‐ended survey responses, documentation, and interview transcripts. Data outputs were compared, and processes of thematic synthesis and triangulation were employed to interpret the combined dataset [13, 14, 15, 16, 17]. See Supporting Material for dataset attributes, analytic processes and digital tools.

For the quantitative analysis, we calculated descriptive statistics across responses to Likert‐type and multiple‐choice questions, which were reported as the number and percentage of each response. Due to low participant numbers, survey responses were aggregated: ‘strongly agree’ or ‘somewhat agree’ were aggregated as ‘agree’, ‘strongly disagree’ and ‘somewhat disagree’ were aggregated as ‘disagree’. Similarly, the number of ‘often’ or ‘always’, and ‘rarely’ or ‘never’ responses was tallied. ‘Neutral’, ‘I don't know’ and ‘sometimes’ answers were kept as such. In percentage calculations, the denominator was the number of respondents who answered the question (valid percentage). Multiple‐choice responses were recorded as the number of people and the percentage of the total sample marking each checkbox.

Qualitative analysis was informed by Braun and Clarke's [16] six phases of reflexive thematic analysis: (1) Familiarising with the dataset, (2) Coding, (3) Generating initial themes, (4) Developing and reviewing themes, (5) Refining, defining and naming themes, and (6) Writing. Braun and Clarke [16] emphasise flexible, interpretative and reflexive research processes. They also regard researcher positionality and experience as valuable resources, which we believe is particularly relevant in the case of lived experience researchers. We conceptualise themes as actively produced by the researchers rather than ‘domain’ or ‘topic summaries’ that ‘emerge’ [16]. We used a predominantly inductive and realist approach, understood to capture the experiences, meanings and reality of participants [16]. Document analysis was additionally guided by Bowen [14] and combined elements of content and thematic analysis. Content analysis identified the general characteristics of co‐design outcomes within documents [14]; thematic analysis explored the bases for these decisions and patterns across and within participant groups and sites [16].

Familiarisation began with three co‐investigators separately reviewing the qualitative datasets—open‐ended survey responses, documentation and interview transcripts—developing initial codes and themes. Each dataset was coded separately using inductively developed codebooks to support reflexive, team‐based analysis. Concept maps were used to organise interpretations, and reflexive diaries were kept during interviews and transcription [16]. The team collaboratively reviewed and refined the codebooks, generating and validating themes, noting exceptions, and describing tensions [16]. Datasets were re‐reviewed for consistency before the codebooks were mapped and merged into a final version for discussion with the wider team.

Quantitative and qualitative data were then integrated. Related constructs from the survey were compared with corresponding qualitative themes [13, 14, 15, 16]. Using convergent triangulation, we explored how data aligned or diverged to inform the synthesis of final themes [16, 17]. Integration and interpretation were guided by lived experience perspectives and collaborative discussion. The results reflect iterative, reflexive team discussions in which meta‐inferences were developed by comparing patterns across datasets [16].

## 3 Results

### 3.1 Survey and Interview Data

We recruited 14 survey participants, the majority of whom (64%) had a role in safe space co‐design as a mental health service user. Survey participants also identified as carers (14%) or paid workers (14%), or held ‘other’ roles (7%). Regardless of their formal role, all but one reported lived experience as a service user, carer or both. Most interviewees (75%) participated in co‐design as a lived experience representative. The remaining two (25%) identified as a steering committee member and a core project team member, respectively.

### 3.2 Documentary Data

There was considerable variability in co‐design processes across the six safe space sites. All sites undertook lengthy and dedicated co‐design processes that engaged service users, carers, peer workers, and health and community professionals, with one exception, which had minimal input from lived experience representatives due to funding being conditional on an extremely short operational time frame. Three of the six sites engaged external facilitators. The scope of local co‐design activities extended to developing key values, principles and ideas for what the safe space environment and experience should look and feel like. Co‐designed outcomes were appraised against parameters set by local steering groups and/or government health guidance documents. Where the implementation and management of safe spaces was devolved to not‐for‐profit organisations, co‐design took place either before or post tender. Formal post‐implementation co‐design was undertaken at two sites to identify priorities for continuous quality improvement.

A key outcome of co‐design was the development of a service model centred on the concept of warm, welcoming and safe non‐clinical spaces where people can openly discuss their distress and suicidality with peers who share a lived experience of suicide [1, 18]. Creating an environment in which guests felt welcomed and comfortable was a central focus, leading to careful consideration of accessibility (location, opening hours and transport) and the physical environment (layout and décor), including the need for quiet areas, sensory rooms and spaces for private and group discussions.

A guiding principle of safe spaces is that they should be open to everyone. Yet, co‐design documentation acknowledged several unresolved challenges. For example, co‐designers had differing views on a full open‐door policy in relation to guests experiencing issues such as social isolation, alcohol or other drug use, or serious and persistent mental illness who wished to access the service (without concern for suicide). These tensions resurfaced during implementation, especially regarding the safe space being used as a ‘drop‐in space’ by some, and the role of peer workers in supporting guests experiencing acute suicidality.

Staffing by an experienced peer workforce was also a key focus in the co‐design documentation. The requirement for peer workers to have lived experience of suicidality and recovery was noted at some sites, while others acknowledged lived experience of distress—particularly the overcoming of distress—as the basis for providing intentional support. A mix of genders, sexualities and cultures that were able to meet community needs was also highlighted. Beyond creating a welcoming environment, co‐design documents focused on the role of safe spaces in meeting guests' support needs, for example, through safety planning, building new skills or capabilities, engaging with professional or informal supports, or warm connections to other services.

### 3.3 Theme 1: Genuine Co‐Design Is Safe, Respectful, Accessible and Inclusive

As shown in Table 1, 100% (*n* = 13) of respondents to the question agreed that participation was a positive experience.

**Table 1:** Survey participants' feedback on co‐design processes.

Participants' process feedback	Numbers			Percentages		
	Often/Always	Rarely/Never	Sometimes	Often/Always	Rarely/Never	Sometimes
Participation was a positive experience for me	13	0	0	100	0	0
Participation was a negative experience for me	0	13	0	0	100	0
Participation had positive impacts on my mental health	12	2	0	86	14	0
Participation had negative impacts on my mental health	0	0	13	0	0	100
I experienced personal distress or triggers during participation	0	2	11	0	15	85
I would plan for extra self‐care after participation	3	4	6	23	31	46
**Participants' process feedback**	**Agree**	**Disagree**	**Undecided**	**Agree**	**Disagree**	**Undecided**
The co‐design group meeting arrangements were accessible for me, for example, meeting times and locations, frequency, time commitment, and accessible location	13	1	0	93	7	0
I felt heard and respected	13	1	0	93	7	0
I felt safe and included	12	1	0	92	8	0
Language and communication were respectful	12	1	0	92	8	0
Sensitive subject matter was discussed in a safe way	13	1	0	93	7	0
Power dynamics were balanced and fair	10	2	1	77	15	8
Decision‐making was balanced and fair	10	2	0	83	17	0
Open access to resources enabled equitable and effective participation of all group members	10	2	0	83	17	0
There was a diversity of membership and perspectives within the group	9	3	1	69	23	8
Updates on the progress and outcomes of the Safe Haven have been relayed back to the group in a timely manner	8	3	0	73	27	0
I am familiar with how the Safe Haven/Safe Space model was implemented in practice	11	2	1	79	14	7
**Benefits of participating**	**Numbers**			**Percentages**		
Sense of achievement	9			64		
Meaning and purpose	9			64		
Contributing my skills to a new project	11			79		
Using my lived experience for the greater good	13			93		
Positive relationships, working together on a shared project	10			71		
Other	1			7		

Participants were ‘very complimentary of the processes’ [Dave], generally finding them safe, respectful, inclusive and balanced. Feedback on accessibility was largely positive, with most respondents (93%, *n* = 13) agreeing that meeting arrangements were accessible. Qualitative comments highlighted helpful facilitation, clear communication and effective collaboration tools. Meetings were well‐organised, with advance scheduling, manageable time commitments and structured formats that supported meaningful participation. When asked what was especially good about the processes, participants often cited ‘respect’ and ‘collaboration’; a majority (69%, *n* = 9) agreed their co‐design group was diverse. Other helpful provisions were comprehensive briefing papers, in‐person visits to assess potential sites, and resource availability, such as having access to other safe space models and approaches rather than trying to innovate from a blank canvas. When asked to choose the most positive things about participating in co‐design, the majority of participants (93%, *n* = 13) said it allowed them to use their lived experience for the greater good.

### 3.4 Theme 2: Power Dynamics, Big and Tricky Feelings, and Not Feeling Less‐Than

Engaging with multiple stakeholder perspectives was described as both valuable and challenging. Many participants felt genuinely included and respected, noting that they were listened to and heard. A majority (93% *n* = 13) of survey respondents agreed that sensitive subject matter was discussed in a safe way and that power dynamics (77%, *n* = 15) and decision‐making (83%, *n* = 17) were balanced and fair. Participants affirmed co‐design processes that did not elevate formal education and qualifications—specifically health and clinical—over lived experience expertise: Ann ‘never felt less‐than in those conversations’, and Mark described being ‘treated as a complete equal from the Director of Mental Health’.

However, for some respondents, power dynamics were a source of frustration. Ann recalled:

I do remember saying, ‘so, the clinical person still has the last say’. That irked me … I had to vocalise my disapproval … the power imbalance … is bloody awful.

Participant 1 (P1) explained:

One of the things that I think is never done very well … is discussions of power … they absolutely take time, and in principle you should be talking about power in a co‐design process for as long as the least powerful people in the process want to talk about it—but that's never how it plays out…. We're actually talking about lives and feelings and experiences, and a lot of that can be very big and very tricky, and not … seen as particularly helpful when you're actually output focused.

Some co‐designs attempted to manage power dynamics by employing a lived experience facilitator, ‘…who was, I think, better at navigating that kind of complexity’ [P1]. Others began with separate clinical and lived experience groups before coming together as a larger group.

### 3.5 Theme 3: Navigating Clinical–Non‐Clinical Tension in Governance, Risk Planning and Safe Pathways

A notable challenge during co‐design was disagreement over the role of clinical care and governance. For many, being fully community‐led and ‘including lived experience in governance’ was integral to a non‐medicalised approach [Mark]. Despite strong advocacy by lived experience voices for peer‐led safe spaces, participants noted that many safe spaces ‘have come on board with a clinical team’ [Sarah], or adopted models shaped more by clinical than lived experience perspectives.

This had implications for guest support policies and referral pathways, with co‐design documentation at some sites indicating an absence of, or lack of clarity on, protocols for external support and risk management. With a focus on crisis transformation and a corresponding ‘no wrong door’ approach, a key consideration for co‐designers was how lived experience‐informed safety planning, risk management and escalation would take place in an atmosphere of ‘dignity of choice’ for guests. Sarah reflected:

People who are clinically trained … [are] hard‐wired to look for risk … [but] there's risk everywhere … [and] people manage their suicidality every day. Let's not frame our space to be all about that … this is a safe space and there's dignity of choice.

Ann saw this clinical–non‐clinical tension as a challenge ‘in all the work that goes on … like postvention and the SPOT [Suicide Prevention] Outreach Teams … the clinical person still has the last say’. She described the essence of a non‐clinical structure:

People have some control over what they need rather than [being told] … what they need, and I think that's one of the most important things for a person who has experienced mental health issues or suicidal crisis, they don't need … that added [clinical] pressure.

### 3.6 Theme 4: Tokenism and Faux‐Design

According to Participant 1, ‘there's structural ways of limiting people's input … because there's being in the room, and then there's actually being able to meaningfully contribute’. Elements of tokenistic co‐design, or ‘faux‐design’ [P1], were described by study participants as inadequate funding, lack of lived experience input, not allowing enough time, lack of information or transparency, starting the process too late, or finishing too early. Failing to adhere to genuine co‐design practices can undermine trust and reduce engagement. For example, lived experience participants were critical of the approach taken at one site that involved a ‘fake competition’:

The design teams proceeded with the understanding that one of our designs would ‘win’ and be implemented. After presenting our designs we were told that wasn't the case. This damaged trust … because we felt that our time and ideas weren't taken seriously. The lack of information about funding and location limitations also meant we were invested in ideas and approaches that were never going to be taken up, which further damaged our trust.

Survey Respondent

Participant 1 described how a lack of transparency can affect a co‐design process:

It's really hard for us to come up with something that is practical and can be implemented if we don't know things like where you *won't* put it … constraints on location … constraints on funding … but we just kept being told, ‘oh no, no, no, forget about that, basically, pretend there's a bottomless bucket of money and, you know, all the people in the world and, you know, pretend that's no object’ … which is just, well, it's not true!

A co‐design facilitator explained how one health service began designing a safe space before the co‐design sessions: ‘some very significant decisions’ were made before lived experience involvement, which ‘discouraged suggestions and made the process seem like tokenism’ when peer workers informed some co‐design participants that their proposals ‘would or would not be happening based on design processes that were happening in their workplace’.

### 3.7 Theme 5: Mixed Fidelity to Co‐Design Outcomes in Implementation

To explore fidelity to the outcomes of co‐design, participants were asked about their satisfaction with service implementation (Table 2).

**Table 2:** Participant satisfaction with implementation of co‐design outputs.

Component	Numbers			Percentages		
	Agree	Disagree	Undecided	Agree	Disagree	Undecided
Location	9	4	1	64	29	7
Opening hours	8	4	2	57	29	14
Physical environment	9	3	2	64	21	14
Therapeutic environment	9	1	4	64	7	29
Culture/vibe	8	3	3	57	21	21
Staffing mix	9	2	1	75	17	8
Policies and procedures	4	3	5	33	25	42
Governance	4	3	3	40	30	30

Participants reported that ‘the majority’ of their group's co‐design ideas were incorporated [Bianca]. For example, a key objective for several groups involved the crafting of the physical or therapeutic environment. Many participants insisted on a warm and welcoming space where compassionate care is provided in a non‐clinical environment, so ‘you're not walking through a swinging door or sliding door, you're not meeting someone with a stethoscope around their neck … [there's] not a lot of forms to fill in …’ [Ann]. Most respondents agreed that this non‐negotiable feature was implemented effectively in terms of the physical space and therapeutic environment.

However, respondents also reported that financial constraints restricted fidelity to key co‐design outputs during implementation. For example, several participants indicated that their co‐design groups preferred more than one site, ‘and we got one trial site’ [P1]. Moreover, 29% of survey respondents and 7 out of 8 interviewees were dissatisfied with safe space opening hours: ‘the reason you go to the emergency department is because other services are closed … consumers … want it after hours or during the “Witching Hour”’ [Bianca], ‘that awful, middle of the night, worst time for a lot of people’ [P1]. They argued that current opening hours leave ‘no option but to go down the emergency medicine route … or to have someone call the police on them’ [P1].

Despite a lack of fidelity to some important co‐design outputs, many participants concluded that having ‘imperfect’ safe spaces was better than not having safe spaces at all. Several participants expressed gratitude that safe spaces existed—‘there wasn't anything and now there is something … it's a change in the right direction’ [P1]. Now in her 70s, Ann has been ‘waiting for these changes for … more than half a century’.

### 3.8 Theme 6: Bridging the Gap: From Co‐Design to Co‐Implementation

Safe spaces are pioneering innovative approaches, and their implementation can be complex, particularly when co‐design processes are involved. Study participants reflected that when co‐designers were not actively involved beyond the design phase, the gap between design and mobilisation disrupted the translation of co‐design outputs. For example, incoming safe space manager, Sarah, received a co‐design document during the contracting process; however, ‘…all those little conversations … what's important, what people value … get lost on the piece‐of‐paper [when] transfer[red] out to a tender or contract’.

Excluding co‐designers from the implementation phase also had repercussions for participants. For P1, a lack of visibility after the design teams finished led to feeling ‘disenfranchised after the fact’. Sonia also felt disenfranchised when ‘all the chief executives of this and that and everything else’ were invited to the official opening, ‘but those of us that were there and had something to input into it … weren't invited.’

Embedding mechanisms for lived experience input into implementation processes and service development was seen as possible. As one participant indicated,

They have to get the services running, and then can continuously contribute to the quality and improvement through co‐design processes … [it's] something that I see in the for‐profit space all the time … piloting … these services and new models of care … and designing them as they go, being agile about that.

Participant 2

Sarah met with co‐designers in the months before the safe space opening. Two years on, she and her team still referenced the co‐design plan in their decision‐making, but she worried, ‘…if I was to move on, would the next person do that?’ At another site, the lived experience peer workers continued co‐designing within their space. Sarah and others suggested that funders need to build provision for continuing co‐design post‐implementation into contracts.

## 4 Discussion

This study highlights challenges and opportunities in implementing genuine co‐design principles and practices across six safe space models. The findings underscore the importance of managing power dynamics, avoiding ‘faux‐design’ and extending co‐design into implementation. A key challenge identified by participants was the imbalance of power between health professionals and people with lived experience. This imbalance can undermine meaningful integration of lived experience into the design process [3, 19, 20]. Time and funding constraints can also restrict meaningful participation [19, 20]. Conducting co‐design in healthcare is particularly difficult as initiatives are frequently undertaken with limited funding and within bureaucratic systems, leading to adaptation or elimination of key project stages to meet time or budget constraints [3, 5, 10]. As we observed, these challenges can lead to the modification or exclusion of important project stages, resulting in brief consultations that minimise the input of lived experience representatives [3, 20]. The interaction between power dynamics and resource constraints can exacerbate these issues. Power‐sharing in authentic co‐design can be managed through flexibility, open communication and shared leadership [10, 19, 20]; however, financial constraints can reduce transparency and amplify power imbalances.

Previous literature, including studies from a diversity of health systems such as Canada, the United States and the United Kingdom, reports that internationally, co‐design is perceived as tokenistic and insincere when decision‐making power sits primarily with health professionals [5, 19, 21, 22]. Similar to our findings, cross‐system reviews demonstrate that budget constraints often force compromises during implementation, further undermining co‐designed initiatives [3, 20]. As a result, what is labelled as co‐design often becomes an exercise in securing buy‐in rather than achieving true collaboration [21], or what our study participants referred to as ‘faux‐design’. Our findings are consistent with existing evidence that faux‐design leads to frustration, disengagement and diminished trust [5, 19, 21, 22].

To address these challenges, our findings reinforce the importance of adequate funding, sufficient time and practices that prioritise shared leadership, transparency and access to resources [19, 21]. Co‐design processes that valued lived experience and prioritised fair decision‐making were considered pivotal to safe and effective co‐design [19]. When these challenges are poorly managed, co‐design can be harmful and sustain the same hierarchies and bureaucracies that it aims to interrupt [20, 21]. Health service providers, therefore, should assess whether they have the capacity and resources for authentic engagement before attempting co‐design [21].

Financial limitations and clinical–non‐clinical tensions complicated implementation. Our study suggests that resource constraints can cause significant deviations from co‐design plans. For example, a significant disappointment for participants was the safe spaces' opening hours. After‐hours support was a key priority for service users and health professionals and is central to accessibility, according to state and national government reports [8, 9]. To this end, the limited operating hours of the safe spaces in our study reduced the sites' ability to meet the objective of providing an authentic alternative to EDs, which was key to the co‐design of these models of care. Once again, this points towards issues of resourcing, transparency and the constraints imposed upon co‐design by external agencies.

Participants also reported that implementation diverged from original co‐design outputs in terms of governance and risk management policies. Despite a priority for peer‐led, non‐clinical governance, some sites were run by government health services, and although no clinicians worked at the sites, guest safety and risk management were shaped by clinical rather than lived experience perspectives. This divergence stems from a risk‐averse assumption that only clinicians can determine safety [23], despite substantial evidence showing that suicide risk assessments provide insufficient information to guide safety and care decisions [24, 25].

A broader challenge was the disconnect between co‐design and implementation. While service user involvement in health service design is well‐established, collaboration in implementation, or co‐implementation, is considerably less developed. Co‐implementation is increasingly recognised as a crucial extension of co‐design, calling for lived experience input at every stage [19, 26, 27, 28]. However, it is often excluded from project frameworks, restricting service user input to early design stages and missing valuable insights during implementation [5, 26, 27, 28]. We found that formal mechanisms to embed lived experience input across project life cycles were lacking, with design teams typically disbanded after design plans were submitted to funders. This lack of continuity can lead to omissions or misinterpretations of co‐designed outputs during implementation.

These findings underscore the need for sustained lived experience engagement to effectively translate design into practice [27, 28]. National policies highlight their role in bridging the policy–implementation gap [1]. Co‐design should involve service users from design through to implementation and evaluation [20, 26, 27, 28], with genuine co‐design embedding lived experience across planning, implementation and evaluation stages [19, 26, 29]. Failure to embed co‐design principles into implementation risks diluting design outputs. Implementation science emphasises ongoing collaboration with lived experience representatives to maintain fidelity to intended co‐design outcomes [27]. Our study found that a lack of mechanisms for continued involvement left participants feeling their contributions were undervalued. Adequate time and funding for implementation are essential [19]. Funders should embed co‐implementation into project contracts, and safe spaces should support mechanisms—both formal and informal—to integrate lived experience input.

Despite the challenges, participants reflected positively on their involvement in service co‐design processes. Many appreciated the opportunity to help create safe spaces and felt that their needs and contributions were respected. Participants emphasised the value of warm, welcoming and non‐clinical environments, which were successfully implemented at several of the sites studied. The existence of these safe spaces was seen as a significant step forward, offering genuine alternatives for individuals in distress. Participants' gratitude for the existence of safe spaces—even when some design aspects were compromised—reflects the broader success of these co‐design processes, despite their challenges.

## 5 Strengths and Limitations

As the quantitative dataset for this study is small (*n* = 14) and represents only six safe space sites, there are limitations to the inferences that can be drawn. Given the risk of both Type I and Type II errors is high in an underpowered study [30], the results provide descriptive statistics only. The qualitative dataset is also limited by the number of respondents (interviews = 8, surveys = 14); however, this was a relatively homogenous population, and the study has narrowly defined objectives [31]. The mixed‐methods convergent design was chosen to elicit the broadest possible understanding of participants' beliefs and the reasoning behind the beliefs expressed [14]. The use of similar questions within the survey instruments allowed comparison, thematic synthesis and triangulation of qualitative and quantitative data. In this way, we leveraged the strengths of mixed‐methods integration during the design, methods, interpretation and reporting levels [14]. Researcher positionality shaped theme development; lived experience guided interpretation, with reflexive diaries supporting ongoing critical self‐awareness [16].

## 6 Conclusion

The aim of mental health service co‐design is to meaningfully engage service users and health staff with the expectation of higher quality and more democratic and ethical healthcare practices. This study contributes to the growing body of evidence on the impact of co‐design in healthcare, particularly in mental health crisis support and suicide prevention services. Power imbalances between clinical professionals and lived experience representatives are a significant barrier to genuine participation and engagement, often intensified by funding limitations. This study highlights the importance of effectively managing power dynamics, avoiding faux‐design and ensuring sufficient resources to support service user input from design through to implementation.

Despite these challenges, and some lack of fidelity to co‐design plans during implementation due to financial constraints, findings demonstrate that authentic co‐design can be rewarding and meaningful. The successful implementation of safe spaces illustrates how co‐design can significantly improve mental health services by supporting the creation of more accessible, non‐clinical alternatives for people in distress. Future co‐design projects should address the barriers that frequently obstruct lived experience involvement and ensure continuous engagement throughout all phases. Crucially, service providers must critically assess their capacity to undertake authentic engagement before attempting co‐design. Addressing these challenges supports fidelity to co‐design outputs and helps develop services that truly meet the needs of their guests. Future research should explore how authentic co‐design principles can be applied in practice, particularly during the transition from design to implementation, to maximise their effectiveness in local contexts.

## Author Contributions

**Erin J. Oldman:** conceptualisation (supporting), data curation (lead), formal analysis (lead), investigation (lead), methodology (supporting), project administration, visualisation (lead), writing – original draft preparation (lead), writing – review and editing (lead). **Michelle Banfield:** conceptualisation (lead), formal analysis (supporting), funding acquisition (lead), methodology (lead), supervision (lead), validation (lead), writing – review and editing (supporting). **Heather Lamb:** conceptualisation (supporting), formal analysis (supporting), funding acquisition (supporting), methodology (supporting), resources (supporting), writing – original draft preparation (supporting), writing – review and editing (equal). **Erin Stewart:** conceptualisation (supporting), data curation (supporting), formal analysis (supporting), methodology (supporting), visualisation (supporting), writing – original draft preparation (supporting), writing – review and editing (supporting). **Helen Tosin Oni:** conceptualisation (supporting), data curation (supporting), formal analysis (supporting), methodology, writing – review and editing (supporting). **Benn Miller:** conceptualisation (supporting), formal analysis (supporting), methodology (supporting), writing – review and editing (supporting). **Mel Giugni:** conceptualisation (supporting), data curation (supporting), investigation (supporting), methodology (supporting), resources (lead), writing – review and editing (supporting). **Alyssa Morse:** conceptualisation (supporting), data curation (supporting), methodology (supporting), resources (supporting), writing – review and editing (supporting). **Scott J. Fitzpatrick:** conceptualisation (supporting), data curation (supporting), formal analysis (supporting), investigation (supporting), methodology (supporting), supervision, validation (supporting), writing – original draft preparation (supporting), writing – review and editing (equal).

## Ethics Statement

The study protocol and consent procedures were approved by the Australian Capital Territory Health Research Ethics Committee under the ‘Co‐Creating Safe Spaces project’ study (Reference Number 2022.ETH.00043).

## Conflicts of Interest

The authors declare no conflicts of interest.

## Supporting information

Supp_material_Co_design_or_faux_design.

## Data Availability

The data that support the findings of this study are available upon request from the corresponding author. The data are not publicly available due to privacy and ethical restrictions.
