# Supplementary material for: Co‐Design or Faux‐Design? Reflections on Co‐Designing Safe Spaces for People in Emotional Distress or Suicidal Crisis in Australia
Source: Health Expect. 2025 Aug 11;28(4):e70379. doi: 10.1111/hex.70379 (PMC12339907; doi:10.1111/hex.70379)
Supplement: Supplementary file 1 — Supp_material_Co_design_or_faux_design. [file HEX-28-e70379-s001.docx]

Supplementary Material for Journal Article:

**Co-design or faux-design? Reflections on co-designing Safe Spaces for people in emotional distress or suicidal crisis in Australia**

Data Analysis

Dataset attributes, analytic processes, and digital tools are described in Table 1.

**Table 1. Dataset attributes and processing tools**

| **Data collection tool** | **Outcome measure** | **Data attributes** | **Framework or analysis tool** | **Data capture tool** | **Data analysis tool** |
| --- | --- | --- | --- | --- | --- |
| Documentation analysis | Outcomes of co-design and implementation | Qualitative | Reflexive thematic analysis (RTA) [^1^] | Microsoft Excel^a^ | Microsoft Word  Microsoft Excel |
| Semi-structured interviews | Experience;  impacts of co-design process | Qualitative;  audio/video recordings and transcripts | RTA | Zoom video conferencing^b^  Interview recordings transcribed using Zoom and Microsoft Word^a^ | Microsoft Excel  NVivo^c^ |
| Online surveys | Experience;  outcomes of co-design and implementation | Quantitative;  Likert scale and multiple-choice questions | Descriptive statistics [^2^] | Hosted on Qualtrics^d^ website;  Data output generated in Qualtrics;  Aggregated in SPSS Statistics^e^ | Microsoft Excel |
|  |  | Qualitative;  open-response questions | RTA |  |  |

*^a^ Version 365, Microsoft, Redmond, Washington*

*^b^ Version 5.17.11, Zoom Video Communications, Inc, San Jose, California*

*^c^ Version 14, Lumivero, Denver, Colorado*

*^d^ Qualtrics, Provo, Utah*

*^e^ SPSS Statistics version 29, IBM, Armonk, New York*

References

​​1. Braun V and Clarke V. *Thematic Analysis: A Practical Guide*. SAGE Publications, 2021.

​2. Shreffler J and Huecker MR. Type I and Type II errors and statistical power. StatPearls Publishing [Internet]. <https://www.ncbi.nlm.nih.gov/books/NBK557530/> (2023, accessed 19 May 2024).​

Interview Guide

The following questions are designed to gather information about steering group members, partners, and co-design participants’ experiences and perspectives of the co-design process and the implementation of the safe spaces.

1. Tell me about your involvement in the safe space co-design process.

*Probe for: How participant heard about it; came to be involved; role of participant; perspective you are providing*

1. What were your expectations of the co-design process?
2. Do you feel you had the opportunity to contribute your expertise to the co-design process in a meaningful way?

*Probe for: Why, why not. Enablers/barriers (for e.g. meeting arrangements; safety; power dynamics)*

1. Do you feel your contributions were listened to and incorporated into the co-design plan?

*Probe for: feeling heard; involvement in decision-making*

1. What experiences stand out to you as helpful or unhelpful in the co-design process?

*Probe for: Access, communication, attitudes, model of care, staff*

1. Did participating in the co-design process have any impacts on you, either negative or positive?

*Probe for: building relationships; skills; confidence; frustration at process/outcomes; mental health impacts*

1. Do you feel the co-design plan has impacted the development of the safe space in a meaningful way?

*Probe for: clear outcomes e.g. model of care, location*

1. Do you feel the safe space model is currently meeting the objectives and outcomes as set out in the co-design plan?
2. From your perspective, is there anything that could be done to improve the co-design process?

Survey Questions

Start of Block: INTRODUCTION

FULL INFORMATION SHEET TO BE POSTED HERE

I have read the Information Statement and declare that I am aged 16 years or over and I am eligible to take part in this survey.

- Yes
- No

Skip To: End of Survey If I have read the Information Statement and declare that I am aged 16 years or over and I am eligib... = No

I consent to take part in the research

- Yes
- No

Skip To: End of Survey If I consent to take part in the research = No

End of Block: INTRODUCTION

Start of Block: Role and Identity

Which of the following (choose one) best describes your role as a contributor to the Co-design of the Safe Haven / Safe Space?

- Consumer voice - Individual, advocate, or representative
- Carer/family/supporter voice - Individual, advocate, or representative
- Paid worker from a peer identified area
- Paid worker from a clinical or service area
- Paid worker from a policy or management area
- Other __________________________________________________

Do you personally identify as (choose all that apply):

- Someone with a lived / living experience of suicidal ideation or distress
- Caring for or supporting someone with suicidal ideation or distress
- Neither of these
- Prefer not to say

How did you become involved in the Safe Haven / Safe Space co-design process? Choose all that apply.

- I initiated my own involvement
- I was asked by someone else
- I responded to a selection process
- It was part of my paid work role
- Other: __________________________________________________

End of Block: Role and Identity

Start of Block: PERSONAL EXPERIENCE OF THE CO-DESIGN PROCESS

The co-design group meeting arrangements were accessible for me. E.g. meeting times and locations, frequency, time commitment, accessible location.

- Disagree
- Somewhat disagree
- Undecided
- Somewhat agree
- Agree

What worked well with the meeting arrangements?

________________________________________________________________

________________________________________________________________

________________________________________________________________

Briefly describe any difficulties with the co-design meeting arrangements.

________________________________________________________________

________________________________________________________________

________________________________________________________________

Briefly describe if and how people external to the main group were brought in to be involved in any way? E.g., for information, advice, collaborative work?

________________________________________________________________

________________________________________________________________

________________________________________________________________

These statements consider communication, decision-making and power-sharing in the co-design process. Please indicate the extent to which you agree with each statement.

|  | Strongly disagree | Disagree | Undecided | Agree | Strongly agree |
| --- | --- | --- | --- | --- | --- |
| I felt heard and respected. |  |  |  |  |  |
| I felt safe and included. |  |  |  |  |  |
| Language and communication was respectful. |  |  |  |  |  |
| Sensitive subject matter was discussed in a safe way. |  |  |  |  |  |
| Power dynamics were balanced and fair. |  |  |  |  |  |
| Decision making was balanced and fair. |  |  |  |  |  |
| Open access to resources enabled equitable and effective participation of all group members. |  |  |  |  |  |
| There was a diversity of membership and perspectives within the group. |  |  |  |  |  |

What was especially good about the co-design process?

________________________________________________________________

________________________________________________________________

________________________________________________________________

What could have been done better in the co-design process?”

________________________________________________________________

________________________________________________________________

________________________________________________________________

Please respond to the following items regarding the personal impact of your participation in the Safe Haven Co-design process.

|  | Never | Rarely | Occasionally | A moderate amount | A great deal |
| --- | --- | --- | --- | --- | --- |
| Participation was a positive experience for me. |  |  |  |  |  |
| Participation was a negative experience for me. |  |  |  |  |  |
| Participation had positive impacts on my mental health. |  |  |  |  |  |
| Participation had negative impacts on my mental health. |  |  |  |  |  |
| I experienced personal distress or triggers during participation. |  |  |  |  |  |
| I would plan for extra self-care after participation. |  |  |  |  |  |

| Page Break |  |
| --- | --- |

What have been the most positive experiences from your involvement in the co-design of the Safe Haven / Safe Space? Multiple responses accepted.

- Sense of achievement
- Meaning and purpose
- Contributing my skills to a new project
- Using my lived experience for the greater good
- Positive relationships, working together on a shared project
- Other __________________________________________________

If either the co-design group or your involvement has come to an end, briefly describe how and why this occurred.

________________________________________________________________

________________________________________________________________

________________________________________________________________

End of Block: PERSONAL EXPERIENCE OF THE CO-DESIGN PROCESS

Start of Block: OUTCOMES AND IMPLEMENTATION

Updates on the progress and outcomes of the Safe Haven / Safe Space have been relayed back to the group in a timely manner.

- Disagree
- Somewhat disagree
- Undecided
- Somewhat agree
- Agree

I am familiar with how the Safe Haven / Safe Space model was implemented in practice.

- Not at all
- A little
- Somewhat
- A lot
- Quite a lot

I am satisfied that the outcomes and decisions of the co-design group are reflected in the resulting Safe Haven / Safe Space.

|  | Strongly disagree | Somewhat disagree | Undecided | Somewhat agree | Strongly agree | Don't know |
| --- | --- | --- | --- | --- | --- | --- |
| Location |  |  |  |  |  |  |
| Opening hours |  |  |  |  |  |  |
| Physical environment |  |  |  |  |  |  |
| Therapeutic environment |  |  |  |  |  |  |
| Culture / vibe |  |  |  |  |  |  |
| Staffing mix |  |  |  |  |  |  |
| Policies and procedures |  |  |  |  |  |  |
| Governance |  |  |  |  |  |  |

End of Block: OUTCOMES AND IMPLEMENTATION

Start of Block: FINAL COMMENTS

Please add any final comments. E.g. personal impact, outcomes, thoughts or improvements regarding the co-design process, something we haven't asked you?

________________________________________________________________
